# Identification of LecRLK gene family in *Cerasus humilis* through genomic-transcriptomic data mining and expression analyses

**DOI:** 10.1371/journal.pone.0254535

**Published:** 2021-07-12

**Authors:** Hongyan Han, Xiaopeng Mu, Pengfei Wang, Zewen Wang, Hongbo Fu, Yu Gary Gao, Junjie Du

**Affiliations:** 1 College of Horticulture, Shanxi Agricultural University, Taigu, Shanxi, P. R. China; 2 Department of Biological Science and Technology, Jinzhong University, Jinzhong, Yuci, Shanxi, P. R. China; 3 OSU South Centers, The Ohio State University, Piketon, Ohio, United States of America; 4 Department of Extension, The Ohio State University, Columbus, Ohio, United States of America; 5 Shanxi Key Laboratory of Germplasm Improvement and Utilization in Pomology, Taigu, Shanxi, P. R. China; National Botanical Research Institute CSIR, INDIA

## Abstract

Lectin receptor-like protein kinases (LecRLKs) have been shown to be involved in plants’ responses to various biotic and abiotic stresse factors. *Cerasus humilis* is an important fruit species widely planted for soil and water conservation in northern China due to its strong tolerance to drought and salinity stresses. In this study, a total of 170 LecRLK family genes (125 G-types, 43 L-types and 2 C-types) were identified in the newly released whole-genome sequences of *C*. *humilis*. Furthermore, nine representative *LecRLK* genes in young plants of *C*. *humilis* under varying drought and salinity stresses were selected for qRT-PCR analysis. Our systematic comparative analyses revealed the active participation of these nine *LecRLK* genes in the salt and drought stress responses of *C*. *humilis*. The results from our study have provided a solid foundation for future functional verification of these *LecRLK* family genes and will likely help facilitate the more rapid and effective development of new stress resistant *Cerasus humilis* cultivars.

## Introduction

During the whole life cycle of plants, both biotic and abiotic factors have significant influence on the growth, development and even survival of higher plants during their life cycle. Membrane localized receptor-like kinases (RLKs), together with other signal receptors like phytochromes, act as the first site of signal perception, which allows the plants to communicate between cells and to respond to changes in their environment [1,2]. Lectin receptor-like protein kinase (LecRLK) family is a group of RLKs that have been shown to play several vital roles in plant pathogenic defense, symbiotic association, insect feeding response, innate immunity and different abiotic stress responses, such as low temperature and high salinity [3–14]. LecRLKs were firstly discover and examined in *Arabidopsis thaliana* [15], and were later isolated in a few important food crops, such as *Oryza sativa* [16], *Pisum sativum* [17], *Solanum lycopersicum* [10], *Pyrus bretschneideri* [2] and *Solanum tuberosum* [14].

Based on the lectin domains’ structures, the LecRLK family has been divided into three categories: G-types, L-types, and C-types [18]. G-type LecRLKs, also known as B-type LecRLKs or S-domain RLKs, have α-D-mannose binding bulb lectin domain, an EGF domain and/or a PAN domain [19]. Overexpression of *GsSRK* (encoding a G-type LecRLK) in Arabidopsis boosted seed germination rate, primary root and rosette leaf growth under salt stress conditions [6]. L-type LecRLKs have a legume-lectin protein-like domain [15]. It was reported that over-expression of *Arabidopsis thaliana LecRK-V*.*5* (an L-type LecRLK encoding gene) led to early stomatal re-opening, enabling Arabidopsis to have stronger susceptibility to pathogen infection [4]. As the smallest LecRLK group, C-type LecRLKs possess homologs of calcium-dependent lectin domain [14]. Contrary to the abundance of G types and L types LecRLK in plants, a very low number of C-type LecRLK was confirmed in several genomes (only one or two copies) [20,21].

*Cerasus humilis* is a small woody shrub that is native to northern China [22]. It has been shown to have good tolerance to drought, poor soil fertility, cold winter temperatures, and high soil salinity [23]. During the last 20 years, this fruit species has been widely planted for soil and water conservation as well as land reclamation from coal mines in Northern China [24,25]. Even though several studies have been conducted to decipher the molecular mechanisms of stress tolerance in *C*. *humilis* [23,26–28], the molecular roles of LecRLKs in the stress response in *C*. *humilis* have not identified and reported yet.

Using the high-throughput sequencing technology, the whole genome and several transcriptomes of *C*. *humilis* have been revealed and published [25,29]. In this study, a comprehensive search of the LecRLKs in *C*.*humilis* genome and tissue-specific transcriptomes was carried out. Phylogenetic relationship, chromosomal distribution, gene structure, and gene duplication events were further analyzed with a series of bioinformatic tools. Moreover, nine representative genes encoding 5 G-type, 3 L-type and 1 C-type LecRLK genes were selected for qRT-PCR analysis in plants of *C*. *humilis* under salinity and drought stresses. The results of this study not only will likely help establish a soild foundation for future functional analysis of the *LecRLK* gene family in *C*. *humilis*, and will also help to speed up the targeted breeding efforts for stress-resistant *C*. *humilis* cultivars.

## Materials and methods

### Identification of *chLecRLK* genes in *C*. *humilis*

The assembled genome of *C*. *humilis* (variety ‘Jinou 1’) was uploaded to the Figshare (accessed with https://doi.org/10.6084/m9.figshare.11669673) from the previous study of our lab (Wang et al., 2020) [29]. The protein sequence of *Arabidopsis thaliana* (Araport11) and *Prunus persica* (Genome v2.0.a1) were downloaded from TAIR database (https://www.arabidopsis.org/) and GDR database (https://www.rosaceae.org/).

Two methods were used to generate the maximum number of *LecRLK* gene family members: Firstly, *LecRLK* genes in *Prunus persica* and *Arabidopsis thaliana* were used for local BLASTP in Genome of *C*. *humilis*. Direct search for *LecRLK* genes in *C*. *humilis* Genome was then conducted by using the Hidden Markov Model (HMM) method [2,14]. The seed files in Stockholm format of G-lectin domains (PF01453), L-lectin domains (PF00139), and C-lectin domains (PF00059) were obtained from the Pfam database (http://pfam.xfam.org/). By running an online hmmsearch program (https://www.ebi.ac.uk/Tools/hmmer/search/hmmsearch) and the local hmmsearch software (HMMER 2.3.2), the sequences in that match PF01453, PF00139 and PF00059 were selected. The NCBI CDD online tool was used for the domain verification (https://www.ncbi.nlm.nih.gov/cdd) and final screening of LecRLK family members was conducted by following the above steps.

### Sequence alignment, phylogenetic analysis, physicochemical properties and subcellular localization prediction

Protein sequences of All *C*. *humilis* LecRLKs obtained in the previous step were used to construct the phylogenetic tree. Using the MEGA software (version 7.1) and the parameters were set as follows: NJ method, P-distance model, Pairwise deletion and 1000 bootstrap replicates [14,30]. With reference to the classification method of *Arabidopsis thaliana LecRLK* gene family, *C*. *humilis* LecRLKs (chLecRLKs) were divided into different classes. The molecular weight (MV) and isoelectric point (pI) of chLecRLKs were predicted using online tool ProtParam (https://web.expasy.org/protparam/). The subcellular localization of chLecRLKs was predicted using the Protein Subcellular Localization Prediction website (WoLF PSORT; https://wolfpsort.hgc.jp/) and double checked with CELLO v2.5: subcellular LOcalization predictor (http://cello.life.nctu.edu.tw/).

### Gene structure, motifs, chromosomal distribution and gene duplication of the *LecRLK* genes in *C*. *humilis*

The distribution of introns and exons of the *LecRLK* genes were searched in *C*. *humilis* genome, and a schematic diagram of gene structures was drawn using Tbtools [31]. Online tool MEME (http://meme-suite.org/tools/meme) was used to conduct protein motif analysis, and default parameters were used except the maximum number of motifs for the conserved domain, which was set to 10.

The chromosomal positions of *LecRLKs* were obtained from *C*. *humilis* Genome by using NCBI local BLASTN, and the chromosomal distribution were visualized by using software Tbtools [31]. The multiple collinearity scan toolkit (MCScanX) was then used to analyze synteny and gene duplication event of *LecRLKs* with default parameters and visualized by Tbtools.

### Expression patterns of *LecRLKs* in different tissues of *C*. *humilis*

Transcriptome sequences of *C*. *humilis* were downloaded from NCBI database (https://www.ncbi.nlm.nih.gov/) under accession number PRJNA417674 (fruits), PRJNA420878 (seed), PRJNA684437 (leaf) and PRJNA683804 (root). Profiles of expressed *LecRLK*s in different tissues were then analyzed with Venn’s diagrams tool (https://bioinfogp.cnb.csic.es/tools/venny/index.html) and the heatmaps were drawn based on the FPKM values of all the differentially expressed *LecRLK*s.

### Plant materials, treatments and physiological responses

One year old plants of *C*. *humilis* (cultivar ‘Jinou 1’, 10 cm high) with were grown in pots (15 cm in diameter) filled with peat moss and perlite (1:1) in a greenhouse. With salt stress treatments, 30 plants (10 for each treatment) were subjected to three different NaCl concentrations (0, 120 and 180 mmol/L); With drought stress treatments, 30 plants (10 for each treatment) were put under three different watering regimes (80%, 50% and 25% of the substrate’s water holding capacity). The substrate’s water holding capacity (WHC) were determined as follows: Firstly, the total weight of pot and substrate (Peat: vermiculite: perlite: river sand = 1: 1: 1: 1) were measured; Secondly, water the substrate throughly and measure the total weight again; Finally, the weight gap was defined as the WHC.

After ten days of being under salt or water stress conditions, leaf MDA (Malondialdehyde), SOD (Superoxide dismutase), POD (Peroxidase) and CAT (Catalase) activity measurements were conducted according to Li et al [32]. Proline contents were measured by following manufacturer’s instructions (Jiancheng Biology Research Institute, Nanjing, China). All measurements were repeated three times for further statistical analysis (SPSS 13.0).

### Expression patterns of selected *LecRLKs* in *C*. *humilis* plants under salinity and drought stresses

Roots and leaves RNAs of plants treated different NaCl concentrations and different water contents were extracted respectively using Trizol Reagent kit (Invitrogen) according to the manufacturer’s protocols. RNA extractions were carried out in three biological replicates. The cDNAs were then synthesized using a high-capacity RNA-to-cDNA Kit (Applied Biosystems, CA). Primers sequences of nine selected *LecRLK*s were designed using Premier 5.0 software (PREMIER Bio-soft International, USA) (S1 File). Quantitative real-time PCR was performed following the method described by Mu et al [25]. Relative expression of each *LecRLK* gene was calculated by the 2^−ΔΔCt^ method [33]. Real-time PCR analysis was also performed in three biological replicates.

## Results

### Identification, classification and physicochemical properties of *LecRLK* genes in *C*. *humilis*

**Fig 1:**
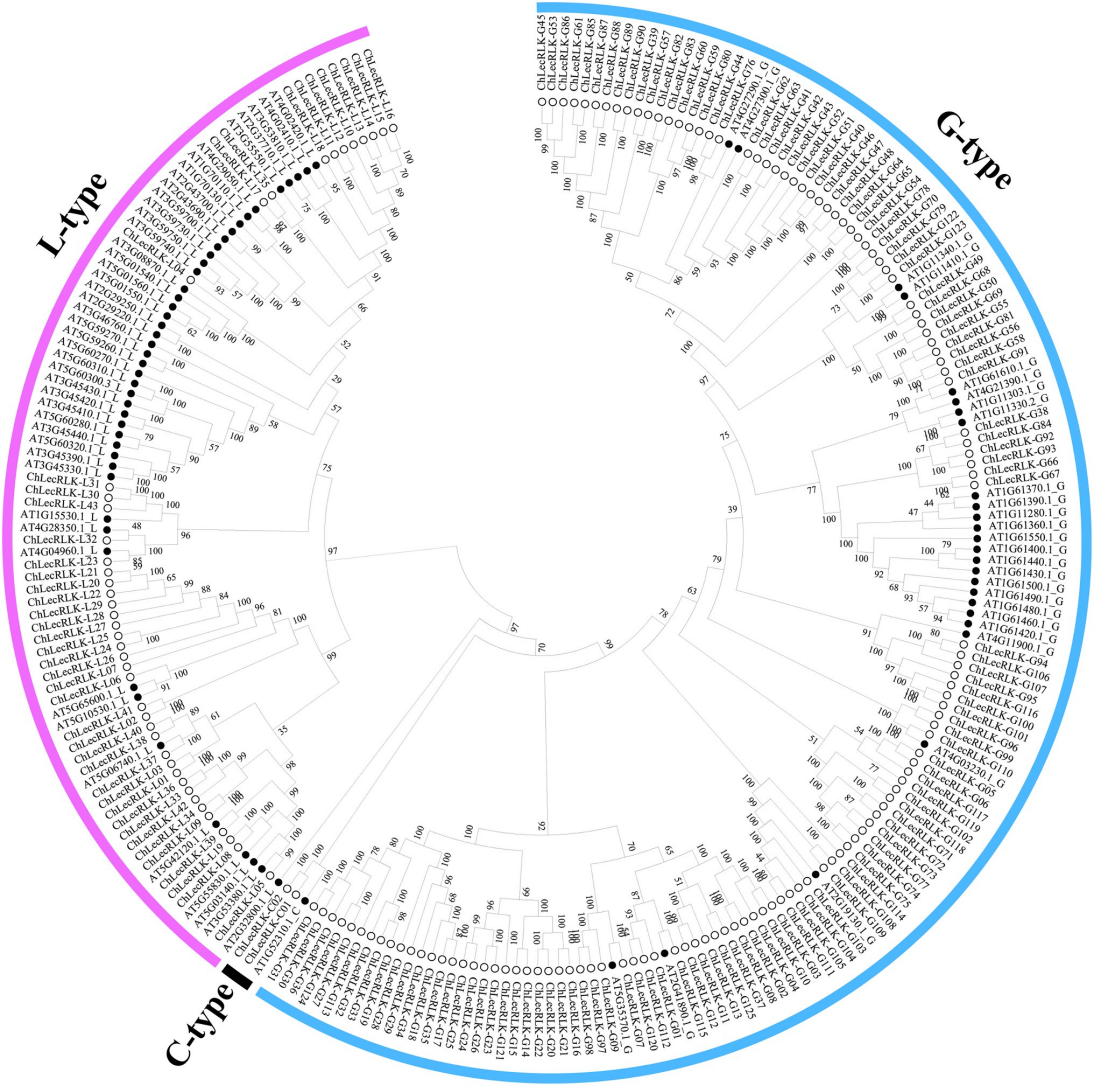
Classification of 170 LecRLKs genes discovered from the whole genome of *C*. *humilis*. The phylogenetic tree was constructed by the neighbor-joining (NJ) method. Solid black dots represent LecRLKs genes of *Arabidopsis thaliana*, hollow circles represent LecRLKs genes of *C*. *humilis*.

Through a database search and subsequent review of the conserved domains, a total of 170 *LecRLK* genes were identified from the *C*. *humilis* genome database, and the phylogenetic tree was constructed using all the LecRLK protein sequences of *C*. *humilis* and *A*. *thaliana* (Fig 1, S1 Table). According to the Arabidopsis classification scheme, the 170 *chLecRLK* genes were divided into three types, which include 125 G-type, 43 L-type, and 2 C-type members.

An analysis of the physicochemical properties of the LecRLKs included the length of the amino acid sequences (aa), the molecular weight (MW), the isoelectric point (PI), and the subcellular localization. Among the 170 LecRLK proteins, the lengths of the amino acid were between 351 and 1627, the MWs were between 38.35 and 180.30 kDa, and the PIs were between 5.01 and 9.56. Although subcellular localization predictions of some chLecRLKs (61 members) were not consistent between WoLF PSORT and CELLO, 63.5% of the chLecRLKs (108 out of 170) were shown to be located on the Plasma Membrane (S2 Table).

### Gene structure, domain structure, chromosomal distribution and gene duplication of the *LecRLK* genes in *C*. *humilis*

**Fig 2:**
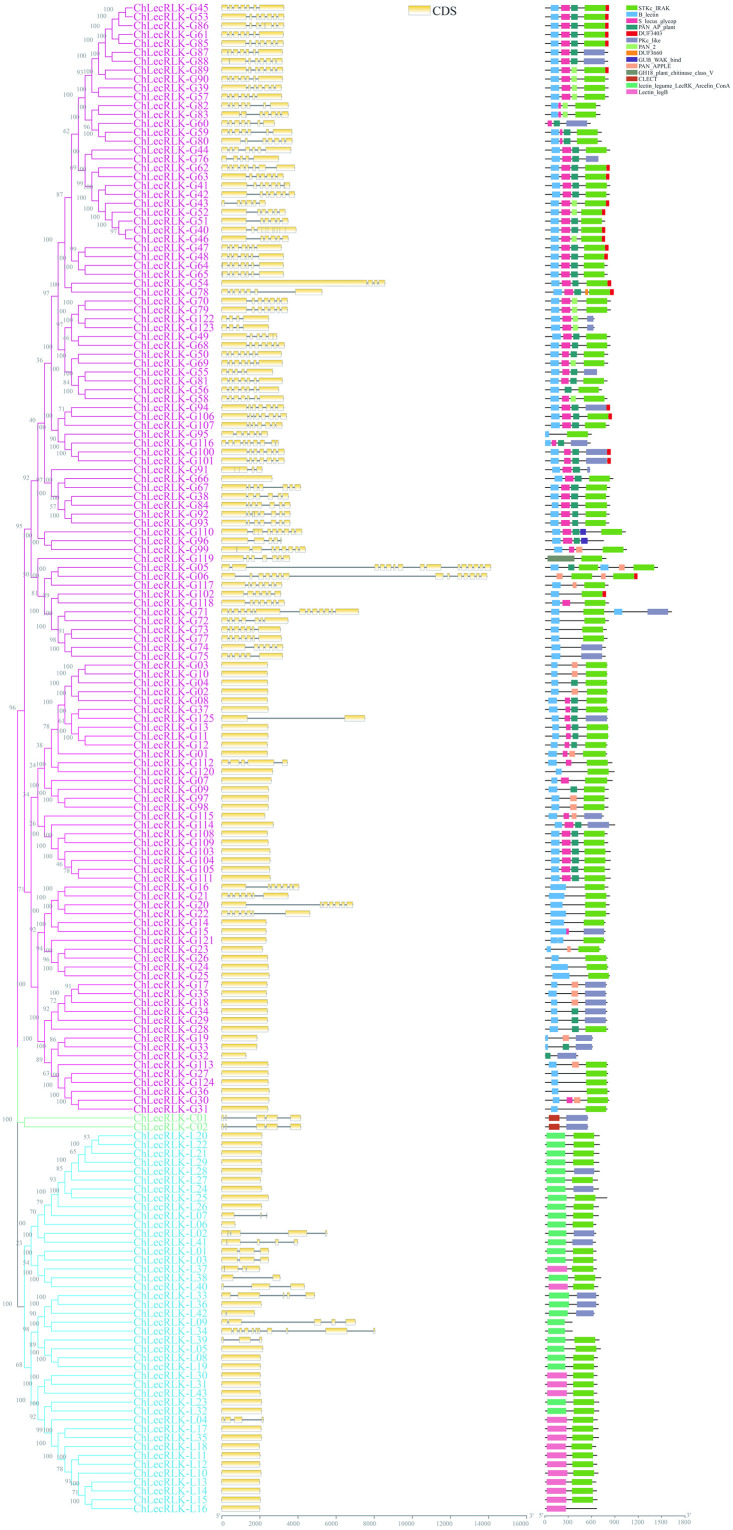
Schematic diagram of the structure of *chLecRLK* genes (left) and conserved domain of chLecRLK proteins (right).

As shown in the schematic diagram of gene structures, exon number of the 170 *chLecRLK* genes ranged from 1 to 14 (Fig 2). Among 125 G-type *chLecRLKs*, 46 family members have only one exon while the rest have at least 4 exons. Most L-type *chLecRLKs* have one exon (29 out of 43), and the *chLecRLK-L34* gene has the most exons (11).

Meanwhile, a total of 14 conserved motifs were obtained from all chLecRLK proteins (Fig 2). Compared with C-type and L-type, G-type chLecRLKs have more complex domains including B_lectin (NCBI CDD: cd00028), S_locus_glycop (pfam00954), PAN_AP_plant (NCBI CDD: cd01098), PAN_APPLE (NCBI CDD: cd00129), PAN_2 (pfam08276), STKc_IRAK (NCBI CDD: cd14066), PKc_like (NCBI CDD: cd13968), DUF3403 (pfam11883), DUF3660 (pfam12398), GUB_WAK_bind (pfam13947), and GH18_plant_chitinase_class_V (NCBI CDD: cd02879). Lectin_legB (pfam00139) and lectin_legume_LecRK_Arcelin_ConA (NCBI CDD: cd06899) domains were uniquely found in L-type chLecRLKs, while CLECT (NCBI CDD: cd00037) was only found in C-type chLecRLKs.

**Fig 3:**
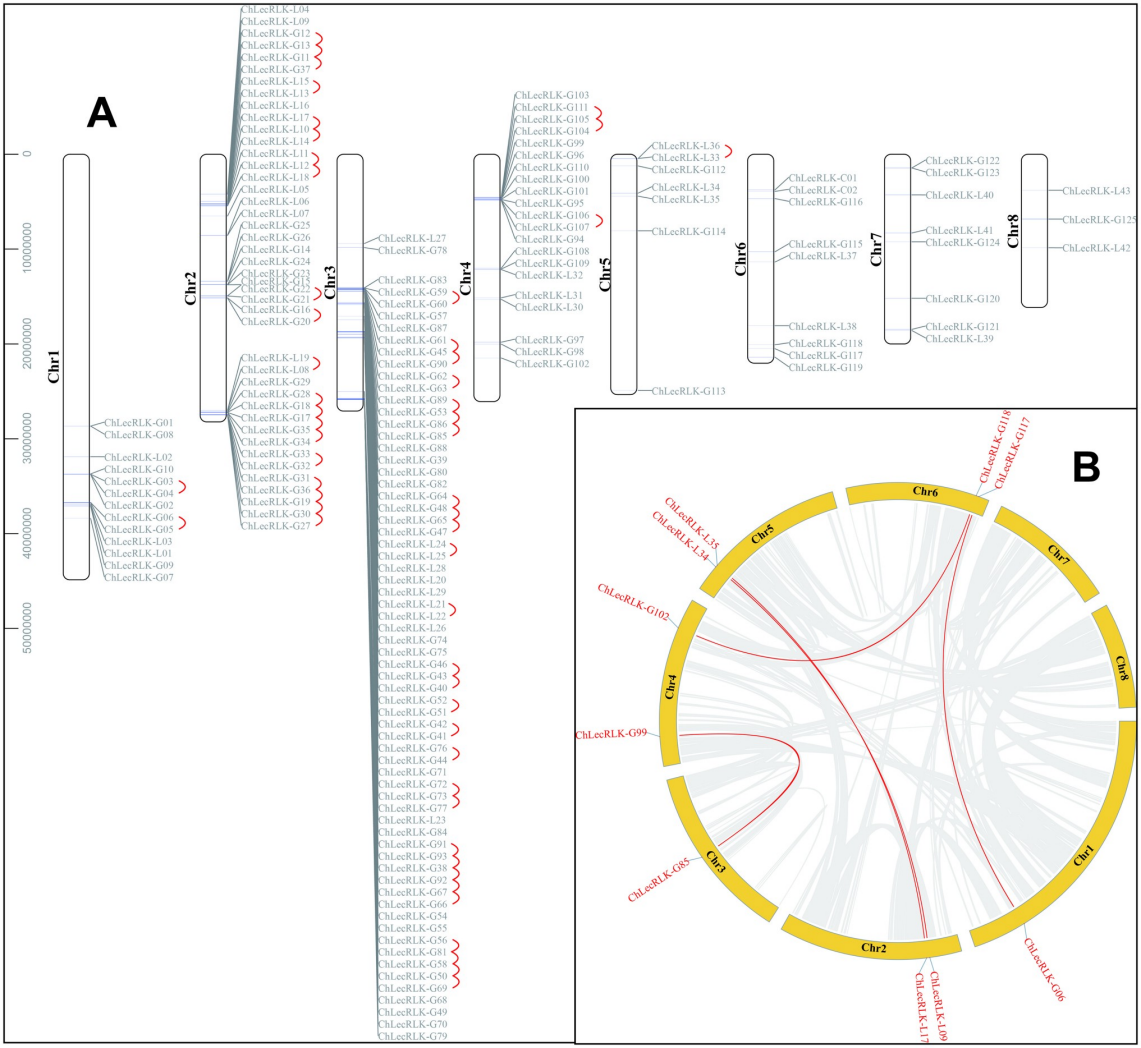
Distribution (A) and synteny (B) of *LecRLKs* genes on eight chromosomes of *C*. *humilis*. Tandem duplicated genes were marked with red curves. Chromosomal distances were given in base pairs. WGD/segmental duplicated genes were marked in red lines.

The *chLecRLK* genes were shown to be unevenly distributed on eight chromosomes (Fig 3A). Chromosome 3 (Ch3) had the most *chLecRLK* genes (56), while only three *chLecRLK* genes were located on chromosome 8 (Ch8). Additionally, gene tandem duplication events (marked in red lines) of *chLecRLKs* were discovered from Chr1 to Chr5, and the tandem repeats contained 83 members consisting of 67 G-types and 16 L-types. Moreover, WGD (whole genome duplication)/segmental duplicated gene pairs were also found containing 6 G-type and 4 L-type *chLecRLKs* (Fig 3B).

### Expression patterns of *LecRLKs* in different organs of *C*. *humilis*

**Fig 4:**
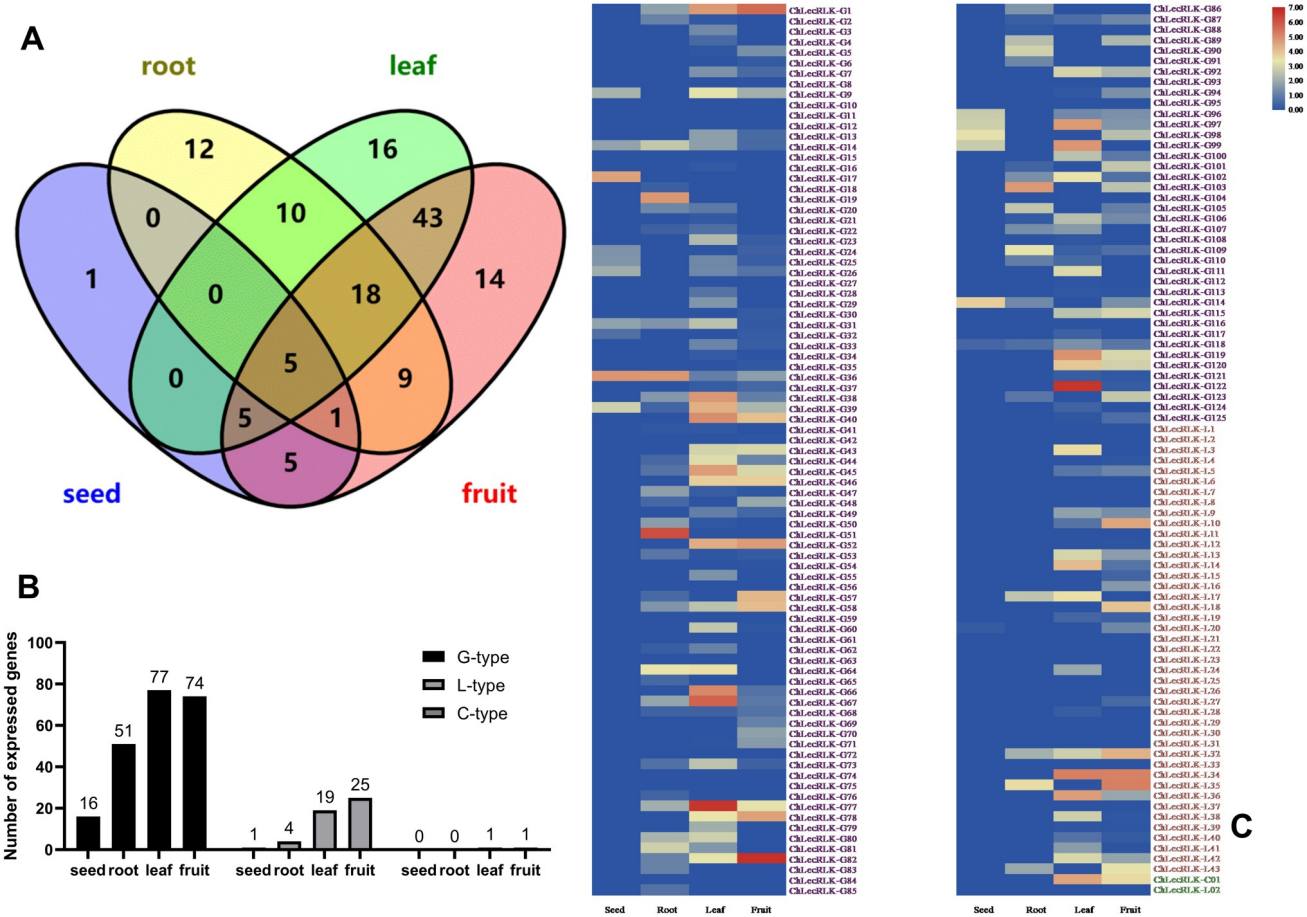
Venn diagram (A), classification (B) and expression (C) of *chLecRLKs* in different tissues of *C*. *humilis*. Names of G-type genes were listed in purple, names of L-type genes were listed in red, and names of C-type genes were listed in green.

Tissue-specific transcriptomic analysis of the leaves, roots, fruits, and seed kernels of *C*. *humilis* were conducted to determine the expression patterns of *LecRLKs* in different organs. In total, 97 and 100 *LecRLK* genes were expressed in leaves and fruits, while only 55 and 17 *LecRLK* genes were found in roots and seeds (Fig 4A, S3 Table). Most of the expressed *chLecRLKs* were G-types, followed by L-types and C -types (Fig 4B, S3 Table).

A heat map of expressed *chLecRLK* genes was generated using the FPKM values (Fig 4C). Most family members of the *chLecRLK* were expressed in one or more organs, and 33 *chLecRLKs* (G-type: 18; L-type: 14; C-type: 1) were not detected in any of the samples examined (S3 Table). Although many *chLecRLK* genes were expressed in different tissues, most of the genes expressed in relatively low amounts (FPKM value < 10) and the genes with the highest FPKM values in different tissues were *chLecRLK-G36* (seed), *chLecRLK-G51* (root), *ChLecRLK-G77* (leaf), and *chLecRLK-G82* (fruit) (Fig 4C).

### Physiological responses of *C*. *humilis* plants to salinity and drought

**Fig 5:**
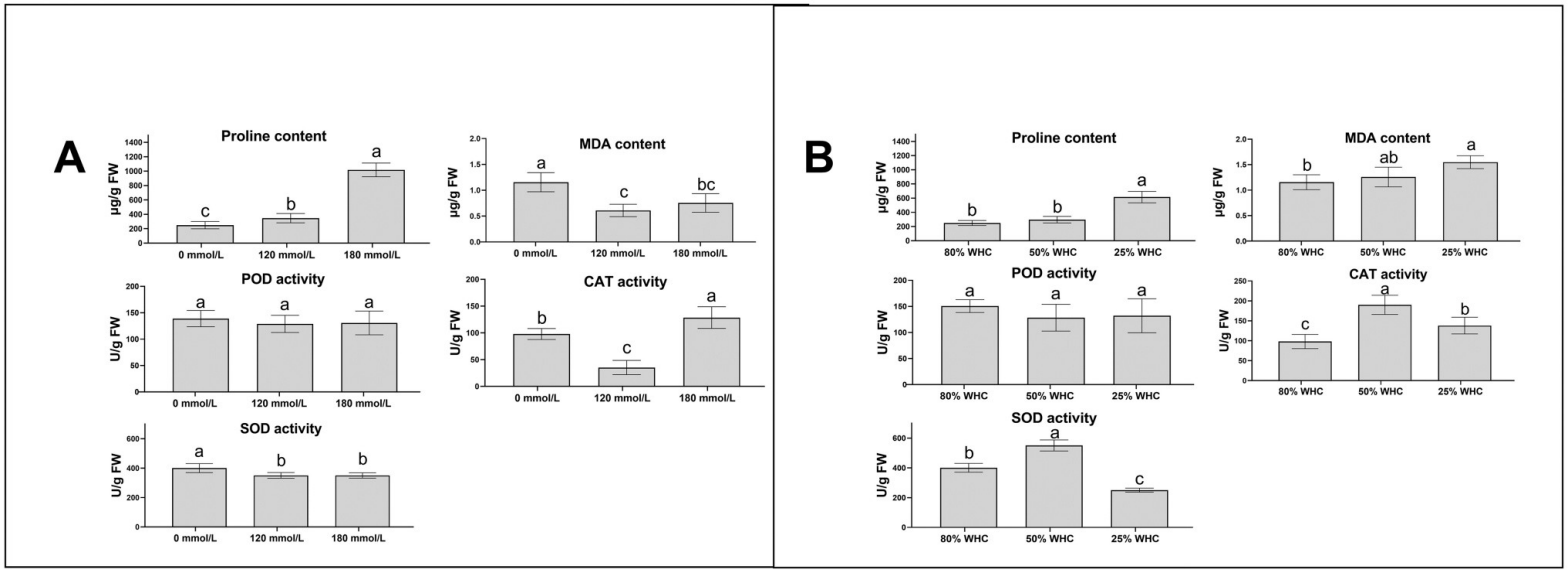
Physiological responses of *C*. *humilis*’ leaves to salinity (A) and drought (B) stresses. WHC represents water holding capacity. Different lowercase letters represent significant difference (p<0.05).

To further validate the effects of salinity and drought stresses to *C*. *humilis* plants, the content of leaf Proline and MDA, and enzyme activities of leaf POD, SOD and CAT were measured. The Proline content increased significantly with the elevation of NaCl concentration and drought while the leaf MDA contents showed different changing patterns (Fig 5). Enzyme activities of leaf POD showed a general decreasing trend in the plants that were subject to both salt and drought stresses, however, this decline did not reach to significant levels. The leaf CAT and SOD activities peaked in plants under light drought stress, but then decreased in plants under severe drought treatment (Fig 5).

### Expression patterns of selected *LecRLKs* in *C*. *humilis* plants under salinity and drought stresses

There were 33 *chLecRLK* genes were commonly expressed in both leaves and roots according to the Fig 4A. These 33 genes were as follows: *ChLecRLK-G9*, *ChLecRLK-G14*, *ChLecRLK-G20*, *ChLecRLK-G22*, *ChLecRLK-G31*, *ChLecRLK-G36*, *ChLecRLK-G38*, *ChLecRLK-G39*, *ChLecRLK-G41*, *ChLecRLK-G44*, *ChLecRLK-G45*, *ChLecRLK-G47*, *ChLecRLK-G50*, *ChLecRLK-G53*, *ChLecRLK-G58*, *ChLecRLK-G62*, *ChLecRLK-G64*, *ChLecRLK-G67*, *ChLecRLK-G68*, *ChLecRLK-G73*, *ChLecRLK-G77*, *ChLecRLK-G80*, *ChLecRLK-G81*, *ChLecRLK-G82*, *ChLecRLK-G87*, *ChLecRLK-G102*, *ChLecRLK-G106*, *ChLecRLK-G107*, *ChLecRLK-G109*, *ChLecRLK-G110*, *ChLecRLK-G118*, *ChLecRLK-L17* and *ChLecRLK-L32*. To further investigate the expression of family members of *chLecRLK* under salt and drought stresses, nine candidate *chLecRLKs* were selected based on their positions and distances between each other on the phylogenetic tree (five G-type *chLecRLKs*: *ChLecRLK-G22*, *ChLecRLK-G36*, *ChLecRLK-G68*, *ChLecRLK-G82* and *ChLecRLK-G107*, three L-type *chLecRLKs*: *ChLecRLK-L17*, *ChLecRLK-L32* and *ChLecRLK-L42*, and two C-type *chLecRLKs*: *ChLecRLK-C01* and *ChLecRLK-C02*) and their expression patterns in leaf and root of NaCl and WHC treated *C*. *humilis* plants were studied.

**Fig 6:**
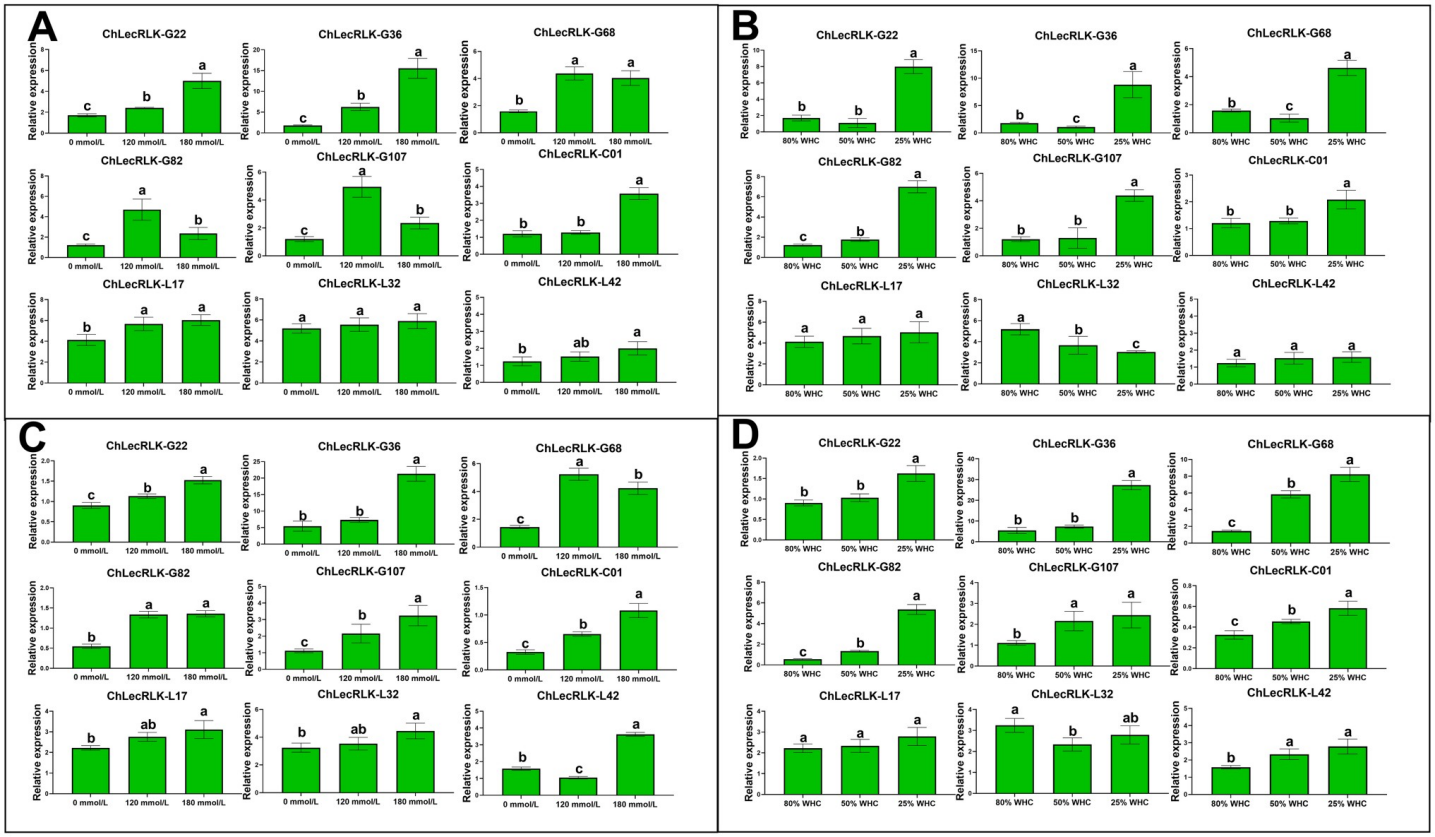
Expression levels of nine members of *chLecRLKs* in *C*. *humilis*. **A**: Expression in leaves under salt stress; **B**: Expression in leaves under drought stress; **C**: Expression in roots under salt stress; **D**: Expression in leaves under drought stress. WHC represents water holding capacity. Different lowercase letters represent significant difference of *chLecRLKs*’ expressions at p<0.05.

The expression patterns of these nine selected *chLecRLK* genes under mild stresses were shown to be significantly different from that under severe stress levels (Fig 6). More specifically, the relative expressions of eight *chLecRLK* genes (5 G-types, 2 L-types and 1 C-types) were significantly elevated in the leaves of plants under severe salt stress (treated with 180 mmol/L NaCl), while only five *chLecRLK* genes (4 G-types and 1 L-types) showed significant increases in expression under light salt stress (treated with 120 mmol/L NaCl) (Fig 6A). Expressions of these nine genes in the leaves of plants under severe drought stress were similar to those under severe salt stress (Fig 6B). However, expression levels of *chLecRLK-G36*, *chLecRLK-G68* and *chLecRLK-L32* decreased in the leaves of plants under light drought stress (Fig 6B).

The relative expression levels of six *chLecRLK* genes (*G68*, *G82*, *G107* and *C01*) were significantly higher than those of the control in roots of plants treated with 120 mmol/L NaCl solutions while the relative expression of all nine genes in roots under 180 mmol/L NaCl treatment were significantly higher than those of the control (Fig 6C). The expression levels of all *chLecRLK* genes were significantly higher in the roots of plants under severe drought stress except *chLecRLK-L32* while only three *chLecRLK* genes (*G68*, *G82* and *G107*) had significantly higher expression levels in roots of plants under light drought stress compared to those of the control (Fig 6D).

## Discussion

### Family size and classification of chLecRLKs

LecRLK proteins are widely distributed in the plant kingdom, and the size of this protein family varies in different plant species. Previous reports showed that *Populus trichocarpa* had the largest LecRLK family of 325 members while *Physcomitrella patens* only had 21 family members [2]. So far, no significant correlation has been found between the sizes of LecRLK family proteins and their corresponding genome sizes [11]. In our study, the number of LecRLK family genes in *C*. *humilis* (170) is similar to that of *Pyrus*. *Bretschneideri* (172) and *Oryza sativa* (173) [2,7].

G-type lecRLK was reported to be the largest subgroup in most plant species [2,11]. One possible theory is that G-type LecRLKs contain more active domains compared to L-types and C-types, therefore possess more biological functions [5]. For example, Special S-locus glycoprotein domain was shown to regulate the self-incompatibility of pollens [34,35]. Epidermal Growth Factor (EGF) domain and/or PAN motif was shown to be involved in disulfide bonds formation, cross-talks between protein to protein and protein-carbohydrate interactions [18,36]. In *C*. *humilis*, G-type chLecRLKs are the major chLecRLKs. Similar results were reported in a few other plant species [11].

L-type LecRLKs are structurally similar to those of the legume-lectin proteins which exhibit glucose-Mannose specificity and they are mostly involved in plants’ responses to biotic stresses [15,37,38]. For example, overexpression of *LecRK-I*.*9* (an L-type lecRLK) in Arabidopsis leads to enhanced resistance to *Phytophthora*. *brassicae* [3]. Another L-type lecRLK encoding gene (*AtLPK1*) is related to elevated resistance to *Botrytis cinerea* [38]. In addition, the L-type lecRLK LecRK-IX.2 not only can activate the immune responses, but also can phosphorylate the bacterial effector AvrPtoB and thereby potentially can reduce its virulence [12,39]. Several reports showed that L-type LecRLKs are also involved in plants’ responses to abiotic stresses stimuli and plants’ developmental processes [40,41].

To date, only one to three C-type LecRLKs were found in different plant species [41]. Only two C-type LecRLKs were found in *C*.*humilis* genome while only one C-type was identified in the rice and Arabidopsis genomes [19]. Unlike G-types and L-types, very few functional studies of the C-type LecRLKs in plants have been published [7].

### Gene duplication and expansion of *chLecRLKs*

Duplication of genes contributes to the functional variations in plants [42]. Previous studies concluded that tandem duplication and WGDs/segmental duplications were the main causes of plant *LecRLKs* [14,43]. In *C*. *humilis*, the amplification of the *chLecRLK* gene family is mainly caused by gene tandem duplications and therefore promotes their evolution. It is worth noting that G-type *LecRLKs* have been more evolutionary expanded compared to L-type *chLecRLKs*, which is in agreements with the findings on *LecRLK* gene family evolution of potato, Taxodium, tomato, pear and Populus [2,11,14].

### Organ-specific expression of *chLecRLKs*

To determine whether the family members of *chLecRLK* in different organs of *C*. *humilis* have special expression patterns, transcriptomic analyses of different organs including seed, root, leaf and fruit of *C*. *humilis* were conducted to determine the organ-specific expressions of all members of *chLecRLK* family. The highest expressed and commonly expressed *LecRLK* genes in four different organs were all G-types suggesting the close involvement of G-type genes in various physiological processes. It is also interesting to note that numbers of expressed *chLecRLKs* in leaf and fruit were much higher than those of in roots and seeds. One possible explanation is that the leaf and fruit are constantly exposed to drastic changes in temperature, light, humidity, and other environmental conditions, more chLecRLK proteins may be needed to “process” external stress signals and their corresponding internal responses, while the roots and seeds are protected by soil and -seed coats respectively, therefore there may not be as big of a need to synthesize a large amount of chLecRLK proteins under relatively more stable environmental conditions. Similar results were also found in two woody plants including *Populus* and *Taxodium* [11,44]. Although having a big family, only a few members may function or express differentially in different organs [11,45].

### Physiological responses and *chLecRLKs* expressions of *C*. *humilis* plants under Salt and drought stresses

Several biochemical indicators including the contents of MDA and proline, activities of SOD, POD and CAT are widely used for evaluating the effects of biotic and abiotic stresses [45]. In our study, the same indicators in *C*. *humilis* plants treated with different NaCl concentrations and watering regimes were measured. The proline content was shown to be the best indicator for evaluating the severity of salt and drought stresses in *C*. *humilis* plants.

To further investigate the potential roles of *chLecRLKs* under salt and drought stresses, qRT-PCR experiments of nine selected *chLecRLKs* were conducted in leaves and roots of the treated *C*. *humilis* plants. The relative expression levels of most *chLecRLK* genes were elevated in the leaves and roots of plants under severe salt and drought stresses thus indicating their participation in responses to severe stresses.

## Conclusions

One hundred seventy *chLecRLKs* genes were identified from the the newly published genome of woody shrub *C*. *humilis*, and they are comprised of 125 G-, 43 L-, and 2 C- types. Gene tandem duplication events were found to be the main cause of expansion of *chLecRLK* genes, mostly G-types and L-types. Numbers of expressed *chLecRLKs* in leaves and fruits were much higher than those of in roots and seeds of *C*. *humilis* based on the tissue-specific transcriptomic analyses. The qRT-PCR analysis of nine selected *LecRLK* genes revealed their active participation in salt and drought stress responses. The results from our study will provide a strong foundation for future functional analysis of *chLecRLK* family genes and will also help facilitate more effective development of new stress-resistant *C*. *humilis* cultivars.

## Supporting information

S1 Table Gene family of *chLecRLKs*. (XLSX)

S2 Table Physicochemical property predictions of chLecRLKs. (XLSX)

S3 Table Tissue-specific *chLecRLKs*. (XLSX)

S1 File Primers for qPCR. (DOCX)
