# Supplementary material for: Identification of LecRLK gene family in Cerasus humilis through genomic-transcriptomic data mining and expression analyses
Source: PLoS One. 2021 Jul 12;16(7):e0254535. doi: 10.1371/journal.pone.0254535 (PMC8274838; doi:10.1371/journal.pone.0254535)
Supplement: S1 File — (DOCX) [file pone.0254535.s004.docx]

| Name | Forward Primer | Reverse Primer |
| --- | --- | --- |
| G22 | GGCCTTACCTTGCTAACAGC | AAGGGTGTCGGTTGGATGAT |
| G36 | ACCCGACAGAACCGTAGTTT | GCTCTTCACCCTGAGGACTT |
| G68 | GCATGGCATGGTTTGGAGAT | ACGCAGCAACGATAAGCAAT |
| G82 | TGAAGCTTGGAGAAGGTGGT | TCCCTGAATGCAGTAGCCAA |
| G107 | AGTCTCAGCAACAGCAGGAT | GTGGTAACCCTGCGTCATTC |
| C01 | TGGACCCAATAAGCGGAAGT | TGAGCAAAGGCCAGTTCTTG |
| L17 | TCGAGCTCTTGACTTGGGTT | TCCAGCTCCCAGGACTCTAT |
| L32 | CCCACTTCCCTTCTCAACCT | AACACCCAAATGCTGAGCTG |
| L42 | AGGAGCTCGCTCATGGAAAT | GACCCTCCTCTTTCCTTCCC |
